# Inequalities in the disruption of paid work during the Covid‐19 pandemic: A world systems analysis of core, semi‐periphery, and periphery states

**DOI:** 10.1111/irel.12310

**Published:** 2022-04-23

**Authors:** Danat Valizade, Manhal Ali, Mark Stuart

**Affiliations:** ^1^ Leeds University Business School University of Leeds Leeds UK

## Abstract

This article reveals the extent of international inequalities in the immediate impact of the COVID‐19 pandemic on participation in paid work. Drawing on World Systems Theory (WST) and a novel quasi‐experimental analysis of nationally representative household panel surveys across 20 countries, the study finds a much sharper increase in the likelihood of dropping out of paid work in semi‐periphery and periphery states relative to core states. We establish a causal link between such international disparities and the early trajectories of state interventions in the labor market. Further analysis demonstrates that within all three world systems delayed, less stringent interventions in the labor market were enabled by right‐wing populism but mitigated by the strength of active labor market policies and collective bargaining.

## INTRODUCTION

The new coronavirus disease—SARS‐CoV‐2 (COVID‐19)—has spurred governments to severely restrict economic activity, which has wreaked havoc on labor markets (Dobbins, 2020; Hodder, 2020; Koebel & Pohler, 2020). Evidence to date suggests that its impact on participation in paid work is deeply unequal (Adams‐Prassl et al., 2020; Brewer & Gardiner, 2020), with significant disparities between developed and developing economies (Ghosh, 2020a; ILO, 2021a). Yet, the employment relations literature has thus far focused mostly on OECD economies (e.g., Adams‐Prassl et al., 2020; Galasso & Foucault, 2020; Koebel & Pohler, 2020; Su et al., 2021). Systematic analysis of the extent and causes of such inequalities in a more global sense is still lacking, a gap we address in this study. Specifically, we ask, have different trajectories of state intervention at the onset of the pandemic led to international inequalities in the likelihood of losing paid work? And, crucially, which factors triggered different trajectories of state intervention in the labor market? To our knowledge, this paper offers the first quasi‐experimental analysis of the impact of the COVID‐19 pandemic on participation in paid work on a global scale.

To address the research questions, we use world systems theory (WST) as a comparative framework (Wallerstein, 1987). This constitutes a novel theoretical contribution to the field of comparative employment relations, which commonly relies on institutional theories (e.g., Varieties of Capitalism) situated within the context of advanced capitalist economies (Fast, 2016; Frege & Kelly, 2013). WST elevates the level of analysis from advanced capitalist economies to three world systems (Chase‐Dunn, 1995; McMichael, 2000; Wallerstein, 1987): core, semi‐periphery, and periphery. The core system comprises wealthy industrialized states with established institutions of employment relations represented by the United States, the United Kingdom, Japan, etc. The semi‐periphery system includes countries transitioning from underdeveloped to developed economies (or vice versa) with emerging labor market institutions, for example, Brazil, India, South Africa, and China. Periphery states are the least developed countries, plagued by weak or non‐existent labor market institutions, informal work and insecurity including Sub‐Saharan Africa and some countries in the Middle East.

Within WST, the world hierarchy is thought to be perpetuated by a political system and established socioeconomic institutions (Chase‐Dunn, 2018; McMichael, 2000). Following this, we posit that trajectories of state intervention in the labor market in the early months of the pandemic were influenced by political populism and the institutionalization of employment relations within world systems. Populism—understood for the purpose of our study in terms of governments or leaders who espouse anti‐establishment and anti‐elitist rhetoric (Acemoglu et al., 2013; Eichengreen, 2018)—matters, because at the onset of the COVID‐19 crisis, when information on the new pandemic was scant, decisions as to whether and when to intervene were to a large extent political (Boettke & Powell, 2021; Gitmez et al., 2020; Milosh et al., 2020; Lipscy, 2020). Right‐wing populism—the currently dominant form of populist ideology (Eichengreen, 2018)—is a growing area of interest for employment relations scholars and the key focus of our study (Cumming et al., 2020). Its aversion to political institutions and science has been consistently linked to the spread of conspiracy beliefs about COVID‐19 and delayed public health interventions (Eberl et al., 2021; Lasco, 2020; Stecula & Pickup, 2021). We hypothesize a similar effect of right‐wing political populism on state interventions in the labor market.

The institutional foundations of labor markets, proxied by collective bargaining and the strength of active labor market policies (ALMPs), understood as measures aimed at placing the unemployed into work, are hypothesized as key predictors of nation‐states’ capacity to protect jobs at the onset of the COVID‐19 pandemic. Our argument stems from a body of work linking collective bargaining with higher trust, coordination, and consistency of government response to economic shocks (Johnstone et al., 2019; Roche & Teague, 2012; Wilkinson & Wood, 2012) and spillover effects from the formal into the informal economy in periphery and semi‐periphery states (Freeman, 2010; Grimshaw & Hayter, 2020; Hayter, 2011; Hayter & Pons‐Vignon, 2018). The strength of ALMPs prior to COVID‐19 is an important factor because they enabled nation‐states, especially core countries, to swiftly expand existing policy instruments or design novel interventions at the onset of the pandemic (Adams‐Prassl et al., 2020; ILO 2021a; OECD, 2020; Stuart et al., 2021). In periphery and semi‐periphery states, where ALMPs are used to mobilize the unemployed and economically inactive, they were leveraged to devise multiple instruments to support informal workers and other insecure groups in the population (ILO, 2021a; Webb, 2021).

Empirically, we use data from the coronavirus government response tracker (OxCGRT) and deploy sequence analysis to construct trajectories of state intervention in the first months of the COVID‐19 pandemic (Hale et al., 2020). These trajectories were merged with microdata from 20 nationally representative, real‐time household panel surveys capturing core, periphery, and semi‐periphery states. This enabled us to deploy a multi‐group interrupted time series (quasi‐experimental) design controlling for time‐invariant individual and state‐level characteristics. The findings revealed a sharp discontinuity in workers’ participation in paid work at the beginning of the pandemic consistent with world systems theory. As hypothesized, within world systems, trajectories of state interventions that caused this effect—especially delayed and weaker interventions in the labor market at the onset of the pandemic—were enabled by right‐wing populism but mitigated by active labor market policies and collective bargaining.

In what follows, we provide theoretical considerations in relation to the comparative impacts of COVID‐19 on employment and justify hypotheses for the present study. Data, measurements, and methods are introduced alongside the outcomes of empirical analysis, followed by a discussion of the theoretical and practical implications of our findings.

## LITERATURE REVIEW AND HYPOTHESIS DEVELOPMENT

### Comparative impacts of COVID‐19 on employment: Theoretical considerations

The defining feature of the COVID‐19 pandemic is that the economic crisis derives from radical restrictions on economic activity rather than the pandemic itself (Guerrieri et al., 2020; Gupta et al., 2020; Koebel & Pohler, 2020). Thus, any international comparative study of the impacts of COVID‐19 on employment ought to draw theoretical links between nation‐states’ interventions in the labor market and the systemic disadvantage of underdeveloped and developing economies in their capacity to support workers throughout the pandemic. This can be problematic, as existing comparative theories (e.g., Varieties of Capitalism [VoC]) are confined both spatially and historically to the advanced capitalist economies (Brady et al., 2011; Ebenau, 2012; Streeck, 2018). Discrepancies between countries are reconciled by the notion of path dependency, assuming convergence of countries toward one of the two modes of market economies (Herrigel & Zeiltin, 2010; Peck & Theodore, 2007). Recent analyses have seen attempts to conceptualize emerging and underdeveloped economies as “informally dominated market economies” (see Dibben & Williams, 2012). However, VoC and other prominent institutional traditions (e.g., varieties of liberalization, the three states of welfare capitalism) remain unconvincing as global comparative frameworks (Ebenau, 2012; Fast, 2016; Muzio, 2021; Streeck, 2016, 2018). Derived from the relational view of the firm, VoC has merit in analyzing firm behavior in different institutional settings. Fundamentally, though, as Streeck (2016: 244) notes, proponents of this approach “make ‘ad‐hoc’ extensions of the standard VoC model to accommodate between‐country differences and abnormalities, thereby protecting the model from falsification.” A comparative study such as ours requires a deeper understanding of the causes of entrenched disparities between developed, underdeveloped, and emerging economies.

Accordingly, we turn to World Systems Theory (WST) as a comparative framework. Associated with the work of Immanuel Wallerstein, the theory formed into an independent school of thought in the 1970s as a response to the then‐dominant modernization theory (Chase‐Dunn, 2018). WST arrives at a taxonomy of world states through the lens of a “multicultural territorial division of labor” (Chase‐Dunn, 1995), which yields the formation of technologically advanced *core states* and subservient *periphery states* whereby the former control the global consumption, distribution of labor, wages, and the transfer of surplus from periphery states (Brady et al., 2011; Chase‐Dunn, 2018; Goldfrank, 2000; McMichael, 2000). WST accepts movement between world systems. Hence, the emergence of semi‐periphery states (transitioning from periphery to core states or *vice versa*) that exhibit some characteristics of developed economies but remain deeply divided into the pockets of rich, industrially advanced urban areas and poor, populous rural areas. The continuing attraction of WST as a comparative framework is that the categorization of nation‐states into the three macro units of analysis—world systems—can accommodate the phenomenon of the new industrial countries (e.g., Singapore and South Korea) alongside emerging market economies (e.g., Brazil, India, and China) while being consistent with inequalities across the north–south divide (Boatca, 2006; Brady et al., 2011).

WST postulates that a stable political system underpinning capitalist relations ensures the dominance of core states, while the extractive nature of political and economic institutions in periphery and semi‐periphery states perpetuates a shadow economy and informal work, making significant swathes of the worker population extremely vulnerable to global shocks (Ghosh, 2020a,b; ILO, 2021a,b). Consequently, global crises hit workers in periphery and semi‐periphery states harder as core states capitalize on the world system to protect their population (McMichael, 2000). The uneven international supply of vaccines, dubbed by Gosh (2021: 3) “the vaccine grab by rich countries,” is an extreme example of how WST manifests in practice.

It follows that at the onset of the pandemic semi‐periphery and periphery states were likely to experience a sharper discontinuity in workers’ participation in paid work than core states (Ghosh, 2020a,b; ILO, 2021a). The true cause of this discontinuity rests with state intervention: public health measures to manage the spread of the virus and labor market support schemes to protect workers’ jobs and income. The economic power of core states enabled them to deploy extensive support measures using short‐ and long‐term job retention schemes to contain the employment and social fallout of the crisis, supporting over 50 million jobs across the OECD economies by May 2020 (OECD, 2020; Stuart et al., 2021). Support schemes at a comparable scale were not available in semi‐periphery and periphery states, which plunged them into an employment crisis before the pandemic really hit them (Ghosh, 2021).

As Ghosh (2020b: 526) notes, “the inadequate spending on relief reflects a wider constraint on the macroeconomic stance.” This is important in that economies in periphery and semi‐periphery states are highly susceptible to sovereign debt ratings, thus limiting fiscal instruments available to them at the beginning of the pandemic (Ghosh, 2020b; ILO, 2021a). According to WST, such inequality is a direct outcome of the world hierarchy where global finances are controlled by core states to maintain their dominant status in the world economy while limiting development opportunities for semi‐periphery and periphery states (Wallerstein, 1987). Hence, support measures in these countries were implemented on a piece‐meal basis (e.g., the “Emergency Program” in Brazil and comparable schemes in India and South Africa) comprising modest transfers of cash and goods as the main means of support for informal workers (Ghosh, 2020b; Webb, 2021).

Consistent with WST, in core states social protection is inseparable from a worker–employer relationship that covers most of the labor market (Frege & Kelly, 2013; Grimshaw & Hayter, 2020; Hayter & Pons‐Vignon, 2018). In periphery and semi‐periphery states where the share of informal workers, migrants with no legal status and the self‐employed can vary between 50 and 90 percent of the working population (Ghosh, 2020b; Grimshaw & Hayter, 2020; ILO, 2021a,b), social protection measures were unable to reach such groups at the beginning of the pandemic (Webb, 2021). That explains the decision to put money directly into people's pockets as the most widely used intervention across Africa, Latin America, and South Asia (Ghosh, 2020b; Webb, 2021). While there is no fundamental reason why periphery and semi‐periphery states could not act resolutely to contain the spread of the virus—indeed, some counties including India and South Africa did implement “draconian” public health measures (Ghosh, 2020b)—the means by which they can support the working population were structurally inefficient. This systemic gap is why we expect semi‐periphery and periphery systems to experience a sharper discontinuity in the share of displaced workers regardless of the trajectory of the disease itself (Ghosh, 2020a,b; ILO, 2021a,b).

These considerations lead to the first two hypotheses.

Hypothesis 1 The effect of COVID‐19 on the likelihood of participation in paid work is significantly more negative in semi‐periphery and periphery systems compared with the core system.

Hypothesis 2 Delayed, less stringent interventions in the labor market in periphery and semi‐periphery systems are associated with a higher likelihood of losing paid work at the beginning of the COVID‐19 pandemic compared with the core system.

### Heterogeneity within world systems: The effect of right‐wing populism

While WST is instrumental in explaining the causal mechanisms behind international inequalities in the effect of the pandemic on participation in paid work, it can mask important heterogeneous effects within world systems. Indeed, the speed and coordination of state interventions in the labor market at the onset of the pandemic varied across developed and developing economies (Adams‐Prassl et al., 2020; ILO, 2021b). Both WST and existing comparative studies of the impact of COVID‐19 indicate that this is likely to be shaped by the political environment and labor market institutions (Adams‐Prassl et al., 2020; Bump et al., 2021).

At the beginning of the pandemic, when governments had to rely on their understanding of a rapidly evolving situation, balancing between public health and economic liberty, decisions as to whether and when to intervene were to a large extent political (Boettke & Powell, 2021; Gitmez et al., 2020; Lipscy, 2020; Milosh et al., 2020). Previous empirical studies have corroborated the decisive role of politics at the onset of the pandemic (Lipscy, 2020; Milosh et al., 2020). In this study, we focus on political populism and, specifically, on right‐wing populist ideology as a dominant form of populism that has two common features: the juxtaposition of “the people” against “the elite”; and an anti‐pluralist rhetoric where populist leaders position themselves as sole representatives of “ordinary” people (Acemoglu et al., 2013; Eichengreen, 2018; Galston, 2018). On a global scale, there is more that unites right‐wing populist leaders than divides them (Eichengreen, 2018; Galston, 2018). Donald Trump (United States), Jair Bolsonaro (Brazil), Viktor Orban (Hungary), and Boris Johnson (United Kingdom), among others, are thought to epitomize right‐wing populist ideology (Eichengreen, 2018).

The inconsistency of the political response to the pandemic in countries with right‐wing populist leaders is well‐documented (Bump et al., 2021; Lasco, 2020; McKee et al., 2020; Stecula & Pickup, 2021). Lasco’s (2020) analysis of the early discourse employed by right‐wing populist leaders in the United States, Brazil, and the Philippines reveal a general pattern toward downplaying the pandemic. This holds true more broadly. As Eberl et al. (2021: 274) note: “The global pandemic almost invites populists to oppose these [public health and economic] measures.” Right‐wing populist leaders derive legitimacy from political division using the pandemic to translate the narrative that will be positively received by their support base (de Andreazzi et al., 2020). This, in turn, impacts the state of employment relations (Budd & Lamare, 2021), with right‐wing populism associated with an overall deterioration in employment practices and the rise of a general climate of insecurity in the labor market (Cumming et al., 2020). These tendencies are likely to have been exacerbated at the beginning of the pandemic.

Hypothesis 3 Right‐wing populism is associated with delayed, less stringent trajectories of state intervention in the labor market at the beginning of the COVID‐19 pandemic.

### Heterogeneity within world systems: The role of labor market institutions

In a global health crisis that wreaks havoc on labor markets, two institutional pillars of labor markets stand out: the strength of collective bargaining and active labor market policies. The influence of trade unions beyond workplace boundaries is well‐documented in the employment relations literature (Budd et al., 2018; Lamare, 2010), particularly at a time of economic crisis (Hickland & Dundon, 2016; Johnstone et al., 2019; Roche & Teague, 2012). It is underscored by the predisposition of collective bargaining to deliberative decision‐making, democratic, cooperative behaviors in the workplace underpinned by trust between employers, employees, and their representatives (Schulz et al., 2021; Wilkinson et al., 2014; Wilkinson & Wood, 2012). Job support schemes during the pandemic were contingent on employers’ cooperation and willingness to partake (Adams‐Prassl et al., 2020; Stuart et al., 2021). This holds for most coronavirus job retention schemes, including those in the United Kingdom, France, Sweden, Denmark, and elsewhere. At the onset of the pandemic, trade unions were exerting pressure on governments to prevent an employment crisis. In the United Kingdom, for example, the Trades Union Congress (TUC) played a key part in the design of the novel Coronavirus Job Retention Scheme, though union involvement in the implementation of the scheme was negligible compared with the role of unions in more coordinated regimes such as Denmark, Sweden, and Germany. Overall, we expect a more cooperative environment with higher collective bargaining coverage to ensure a swifter, more coordinated response in the early months of the pandemic.

Similar mechanisms could have been at play in semi‐periphery states transitioning toward an employment relations system underpinned by institutions of voice and representation (Bhattacherjee, 2001; Cooke, 2009; Erickson et al., 2003; Frege & Kelly, 2013). For instance, in Brazil, the decision to suspend employment without terminating an employment contract was devolved to the workplace level. Yet, in many periphery states, collective bargaining is limited to the formal economy leaving a significant proportion of the workforce in the informal economy unprotected (Grimshaw & Hayter, 2020; Hayter, 2011). However, where collective bargaining is concentrated in sectors crucial for economic prosperity, it can have spillover effects into the informal economy (Freeman, 2010; Hayter, 2011). Unions can influence working conditions within the supply chain providing some degree of security for informal and migrant workers (Hayter & Pons‐Vignon, 2018; Reinecke et al., 2018). During the pandemic, formal sectors with collective bargaining stood a higher chance to recover and in doing so buttress the informal sectors.

ALMPs are another institutional factor affecting trajectories of state intervention in the labor market. ALMPs represent a set of schemes designed to place the unemployed and economically active into work. These might include, but are not limited to, vocational training, assistance in job search and, crucially, employer incentives (e.g., wage subsidies and recruitment assistance). Nation‐states with established ALMPs, particularly those targeting the demand side of the labor market to motivate employer participation, had a sufficient policy base to act swiftly at the onset of the pandemic. Where job protection schemes such as *kurzarbeit* (a social insurance program where employers voluntarily reduce working hours to avoid layoffs) in Germany had been derived from existing ALMPs, employers were more compliant and ready to partake (Adams‐Prassl et al., 2020). Conversely, where a swift extension of current ALMPs had been impractical and novel interventions had to be designed, such as the Coronavirus Job Retention Scheme in the UK, state interventions were delayed and subject to many revisions (Brewer & Gardiner, 2020; Hale et al., 2020).

ALMPs were instrumental in protecting informal workers and those living in poor, rural areas. Efforts to stimulate employers financially in periphery and semi‐periphery states were hampered by a low level of digitization and transparency in private and public finances. ALMPs in such circumstances were implemented to create jobs in the public sector, thereby providing employment opportunities to self‐employed and informal workers displaced by the pandemic (ILO, 2021a,b). The inherent difficulty in supporting informal workers and the self‐employed explains the existence of well over 1,000 jobs programs across periphery and semi‐periphery states (De La Flor et al., 2021; Dhingra & Kondirolli, 2021).

The concluding hypotheses are thus.

Hypothesis 4 Collective bargaining coverage is associated with more stringent trajectories of state intervention in the labor market at the onset of the COVID‐19 pandemic.

Hypothesis 5 The strength of existing active labor market policies is associated with more stringent trajectories of state intervention in the labor market at the onset of the COVID‐19 pandemic.

## DATA AND MEASUREMENTS

### Nationally representative household panel surveys and data on nation‐states’ responses to the pandemic

To understand the immediate impact of COVID‐19, many countries administered real‐time household panel surveys as part of existing nationally representative panels. In the United Kingdom, participants from Understanding Society, the UK household longitudinal study, were asked to complete repeated online questionnaires measuring their welfare, personal circumstances, and employment status during the pandemic. A similar approach was taken in other core countries including the United States (Understanding America Study), the Netherlands (LISS panel), Germany (GESIS panel), and so forth. In semi‐periphery countries, nationally representative household panel surveys continued with high frequency throughout the pandemic: in Brazil, multiple waves of the National Household Sample Survey (PNAD) have been conducted since the onset of COVID‐19; the same holds for South Africa (General Household Survey). In other countries, high‐frequency household phone surveys were conducted spanning the period before and after the COVID‐19 pandemic.

We utilized 20 national household panel surveys representing three world systems: core (the United States, the United Kingdom, Germany, Austria, France, Netherlands, Switzerland, and Finland), semi‐periphery (Brazil, South Africa, and India), and periphery (Malawi, Mali, Uganda, Ethiopia, Burkina Faso, Nigeria, Indonesia, Honduras, and Costa Rica). At the time of writing, these were all the available national household panel surveys for which we could locate pre‐ and post‐COVID‐19 microdata. The sampling frames for these surveys were drawn using stratified weighted samples deemed representative of national households (in semi‐periphery and periphery countries, the samples were further stratified by urban and rural areas). We collected enough data to link individuals across time in all three world systems, thus arriving at balanced panels spanning time periods before and after the onset of the pandemic. This enabled us to estimate the discontinuity with individual fixed effects and standard errors clustered at the country level. We harmonized the surveys to capture five time periods prior to the pandemic (2019 and January–February 2020; where longer panels were available we included data for 2016, 2017, and 2018) and four periods after the start of the COVID‐19 crisis (April–May, June–July, and August–September 2020). Statistical outcomes were weighted to adjust for different sizes of country samples, with a harmonized panel across all countries and time periods spanning circa 1.7 million person‐specific observations. We explain how country surveys were harmonized in the online Appendix.

Data on state interventions were derived from the Oxford COVID‐19 Government Response Tracker (OxCGRT). OxCGRT is a portal that accumulates data on government responses to the pandemic from almost every part of the globe (Hale et al., 2020). The OxCGRT team analyze all available information on state interventions on a daily basis and categorize them into a number of indicators covering a wide range of public health (e.g., school closure, travel bans, and national and local lockdowns), income support, and fiscal measures. OxCGRT derives these variables by taking into account national, regional, and sectoral policies. We used a raw dataset comprised of 108,185 daily observations across 185 countries, covering data from February until December 2020. This is by far the most complete and reliable comparative international database of countries’ responses to the COVID‐19 pandemic.

### Measurements

#### Dependent variable

The dependent variable captures respondents’ participation in any form of paid work, including employed, self‐employed and, crucially, informal workers in periphery and semi‐periphery states. This is a dummy variable where 0 signifies participation in paid work; hence, the regression estimates indicate the probability of losing paid work during the pandemic. The country samples exclude the following: retired people; those in education and/or undertaking professional training; apprenticeships; those unable to work due to a long‐term illness or disability. In all country surveys, those on various types of furlough or job retention schemes were treated as in work with the survey questions formulated accordingly. The measurement of participation in paid work is different from and, arguably, more comprehensive than national labor market statistics on unemployment. This is particularly important for periphery and semi‐periphery states where the formal measurement of unemployment can be misleading due to the high share of informal work and self‐employment. Thus, our dependent variable is efficient at capturing the impact of the pandemic on workers on a global scale (ILO, 2021b).

#### World systems

World systems is a categorical variable with three categories: core, semi‐periphery, and periphery. While within WST debates are ongoing as to the status of some countries (e.g., Eastern European countries, Portugal, and Malaysia), the countries included in our study are broadly representative of the three world systems (Chase‐Dunn, 2018).

#### State interventions

The variable measuring state interventions was derived from the OxCGRT dataset using indicators directly connected to work and employment: workplace closure and income support. The former is effectively a measurement of lockdowns that we recoded into two categories where “0” corresponds to no workplace closure including a “soft” recommendation to work from home; “1” refers to some form of stringency where most or all sectors except essential services were ordered to close. The income support variable has three categories: no income support; government support less than 50 percent of lost income (50 percent of median income if a flat sum); more than 50 percent of lost income (or median income is a flat sum) is covered by the government. The measurement of income support covers all types of schemes including direct cash payment, universal basic income, and its variations as well as payments to employers directly linked to payroll (Hale et al., 2020). The measurement reflects sectoral coverage where support schemes were targeting specific sectors. The OxCGRT includes a dichotomous (0,1) flag to indicate whether all workers including the informal sector were covered or only those in the formal economy. This was important for classifying semi‐periphery (e.g., India) and periphery states where up to 90 percent of workers could be in the informal sector. Using this additional filter, we were able to classify countries that provide substantial support to workers in the formal economy only as no support or below 50 percent support depending on the size of the informal economy. Overall, these measurements are comprehensive indicators enabling comparisons between the three world systems.

To construct trajectories of state response, we created a variable that represents the *daily state of labor market interventions* by intersecting the workplace closure and income support variables. This returned six different states ranging from no workplace closure combined with no income support to workplace closures with more than 50 percent income compensation:

‐ State 1: No workplace closure—no income support (account for 23.0 percent of daily global policy responses in 2020).
‐ State 2: No workplace closure—less than 50 percent income support (10.1 percent).
‐ State 3: No workplace closure—more than 50 percent income support (8.9 percent).
‐ State 4: Workplace closure—no income support (17.2 percent).
‐ State 5: Workplace closure—less than 50 percent income support (19.9 percent).
‐ State 6: Workplace closure—more than 50 percent income support (20.9 percent).

#### Populism

Two datasets were used to construct a measure of populism across the sample of countries: University of Gothenburg's 2020 Varieties of Democracy Dataset; and 2019 Global Party survey. First, the Varieties of Democracy dataset was used to identify the ruling party (or a country leader) for each country by utilizing information on each country's latest election year and the percentage of seat share across the participating political parties. Data on the current ruling party or country leader for each country were then merged with the Global Party Survey (GPS) to obtain the measure of each country's political populism. In the GPS, political populism is conceptualized as a form of discourse or rhetoric making two claims: (1) the only legitimate democratic authority flows directly from the people; and (2) the establishment is the enemy of the people. GPS uses the method of expert surveys to measure party ideological values and positions. Experts were defined as scholars, such as political scientists, who had demonstrable knowledge of the electoral process and parties in a particular country, for example, through publications or membership of a relevant research group. The GPS questionnaire was administered via Qualtrics in November‐to‐December 2019, covering information from 1861 experts on 1043 political parties representing the lower (or single) House of Parliament/Congress from 163 countries. On average, each country included responses from a dozen experts, but the numbers varied a great deal.

The key indicator of populism was measured on 0‐to‐10‐point scale, where 0 indicates *Strongly favors pluralist rhetoric* and 1 indicates *Strongly favors populist rhetoric*. The scale is based on multiple indicators of populism. These include the following: *salience of populist rhetoric*; *will of the people in deciding important issues*; *politicians should lead will of the people*; *corrupt politicians*; *and strongman rule*. In the GPS questionnaire, populist rhetoric is mentioned as language that typically challenges the legitimacy of established political institutions and emphasizes that the will of the people should prevail. In contrast, pluralist rhetoric rejects these ideas, believing that elected leaders should govern, constrained by minority rights, bargaining and compromise, as well as checks and balances on executive power.

The responses are then combined as a single continuous measure of 0–10 and divided into 1–4 ordinal categories, where 1 = “Strongly Pluralist”; 2 = “Moderately pluralist”; 3 = “Moderately Populist”; and 4 = “Strongly Populist.” For the purposes of empirical analysis, the ordinal measurement was then calibrated to range from 0 to 1, where 1 indicates a strongly populist country.

Given the selection of countries for the present study and its timing, the measurement of populism introduced above is effectively a measurement of right‐wing populism consistent with the theoretical rationale for Hypothesis 3. We cannot estimate with the available data how left‐wing populist leaders (e.g., Jeremy Corbyn in the United Kingdom) would have reacted to the evolving global crisis and whether existing labor market institutions would have constrained or amplified their ideological position. This could be considered in future research.

#### Active labor market policies

The strength of ALMPs utilized the sub‐index of the Global Competitiveness Index that measures the strength of active labor market policies. The data for the index are gathered by Executive Opinion Survey as part of the World Economic Forum. The questionnaire asked, on a 7‐point scale, about the extent to which labor market policies help unemployed people reskill and find new employment. The scale was calibrated and converted into an index ranging from 0 to 100.

*Collective bargaining*was measured as a proportion of workers within each country covered by collective agreements (derived from the ILO database).

#### Other covariates

Regression models were controlled for country‐level (GDP, Gini, absolute poverty rate, average broadband speed, COVID‐19 infection and mortality rates, the share of informal economy and self‐employed, employment‐to‐population ratio, mean weekly working hours, and seasonally adjusted unemployment rate) and individual characteristics (occupational characteristics, age, marital status, and household size). Regression estimates on the impact of political populism additionally controlled for proximity to a general election. Descriptive statistics for some key variables, including their means and standard deviations, are reported in Table 1 for each world system.

**TABLE 1:** Descriptive statistics

	Mean	SD	Min	Max	Range	SE
Core countries						
Participation in paid work (1—not in paid work)	0.28	0.45	0.00	1.00	1.00	0.00
Internet speed (mbp/s)	34.11	10.84	19.96	51.54	31.58	0.01
GDP (USD)	53,224.99	12,602.49	42,912.68	77,981.06	35,068.38	15.94
Union density (%)	19.92	10.19	8.03	66.71	58.68	0.01
Collective bargaining coverage (%)	35.65	24.37	12.01	98.25	86.24	0.03
Populism (0–1, index)	0.68	0.20	0.06	0.86	0.80	0.00
Weak early response (%, derived by sequence analysis)	0.08	0.05	0.01	0.15	0.14	0.00
Labor market policies (index, 0–100)	64.44	9.10	55.08	79.22	24.15	0.01
Sample size	624,844.00					
Periphery countries						
Participation in paid work (1—not in paid work)	0.26	0.44	0.00	1.00	1.00	0.00
Internet speed (mbp/s)	2.03	0.77	1.10	3.22	2.12	0.00
GDP (USD)	1062.23	752.66	513.03	2430.14	1917.11	3.50
Union density (%)	7.63	1.61	5.50	8.85	3.35	0.01
Collective bargaining coverage (%)	12.83	4.00	9.80	18.10	8.30	0.03
Populism (0–1, index)	0.50	0.26	0.07	0.78	0.70	0.00
Weak early response (%, derived by sequence analysis)	0.87	0.21	0.46	1.00	0.54	0.00
Labor market policies (index, 0–100)	26.13	5.83	18.53	33.16	14.63	0.03
Sample size	46,123					
Semi‐periphery countries						
Participation in paid work (1—not in paid work)	0.55	0.50	0.00	1.00	1.00	0.00
Internet speed (mbp/s)	6.74	0.24	6.70	8.29	1.59	0.00
GDP (USD)	10,978.89	925.73	1970.44	11,124.06	9153.62	0.50
Union density (%)	17.87	1.72	12.80	28.90	16.10	0.00
Collective bargaining coverage (%)	64.99	5.15	31.13	65.78	34.65	0.00
Populism (0–1, index)	0.94	0.05	0.55	0.95	0.39	0.00
Weak early response (%, derived by sequence analysis)	1.00	0.10	0.85	1.00	0.15	0.00
Labor market policies (index, 0–100)	35.23	15.12	21.19	43.16	21.97	0.05
Sample size	1,054,111					

## ANALYTICAL STRATEGY

**FIGURE 1:**
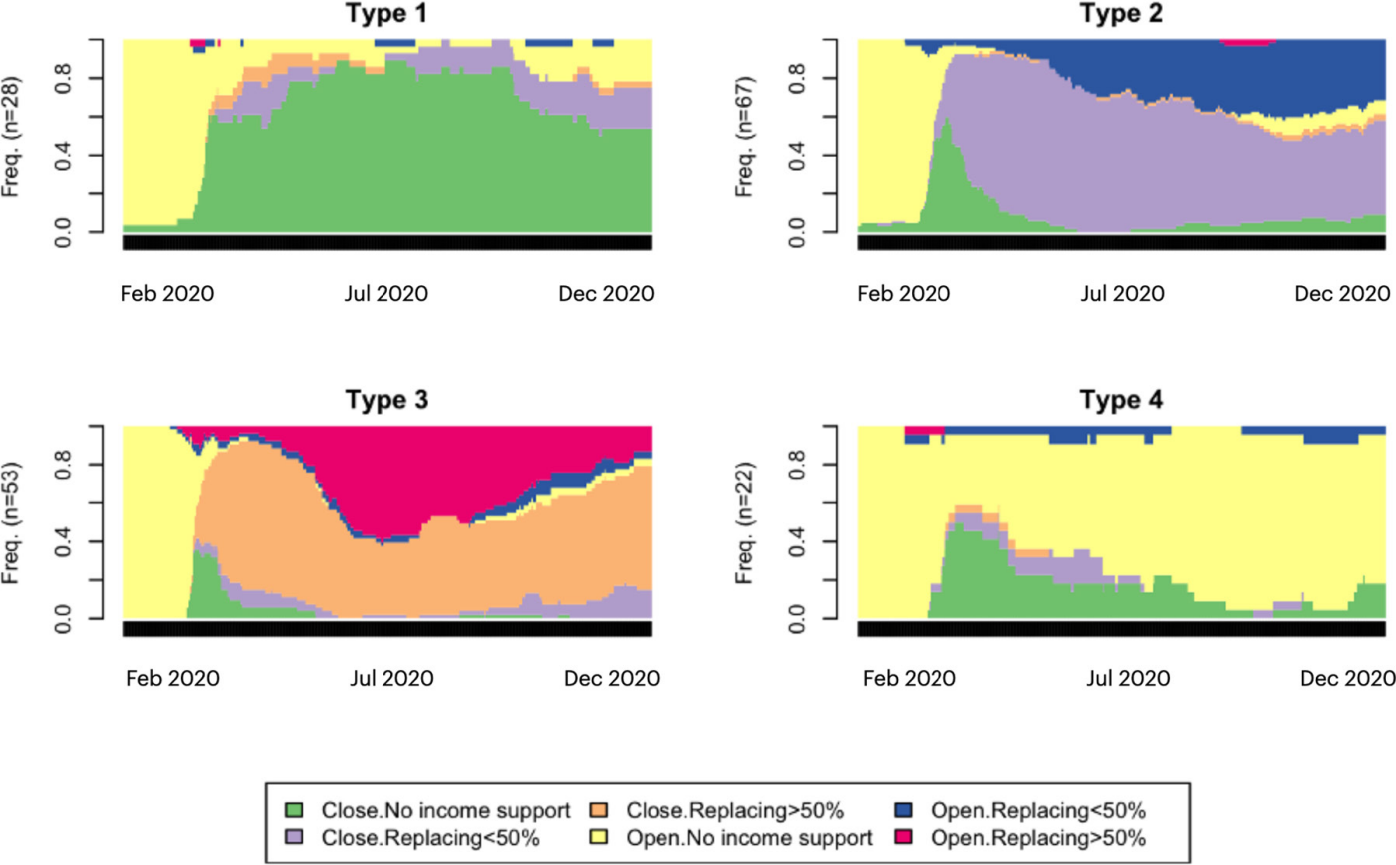
Clusters of state interventions in the labor market

The first step in our analytical strategy was to establish trajectories of state intervention in the labor market by sequencing the six states of government response to the pandemic outlined above. Sequence analysis mimics DNA sequencing and treats each data point in a time series sample as an objective state rather than a stochastic process (Abbott & Tsay, 2000). Applied to the OxCGRT data, sequence analysis returned ordered lists of states over time demonstrating how state interventions changed throughout the pandemic. In the online Appendix, we report raw sequences for all nation‐states from the beginning of the pandemic through to December 2020. Using country‐specific sequences, we estimated dissimilarities between trajectories of state intervention using an optimal matching procedure (Abbott & Hrycak, 1990) and classified countries into subgroups using hierarchical (Ward) clustering. Figure 1 illustrates the emerging four clusters of nation‐states. Cluster 1 characterizes nation‐states where strict public health measures were in place at the onset of the pandemic but without explicit income support schemes (the cluster includes some semi‐periphery countries including Brazil, Mexico, and India and several periphery countries in Sub‐Saharan Africa). Cluster 2 represents countries that used a mixture of robust interventions with support packages covering less than 50 percent of the average pre‐pandemic income (e.g., South Africa, Chile, Easter Europe, and Portugal). Cluster 3 is characterized by strong support measures throughout the pandemic covering more than 50 percent of the average income (most of the core states including continental Europe, the United Kingdom, and the United States). Lastly, cluster 4 includes countries with no compulsory workplace closures (except at an early stage where some nation‐states had deployed strict public health measures) and the absence of national support measures (mostly periphery countries in Sub‐Saharan African, North Africa, and Middle East).

**FIGURE 2:**
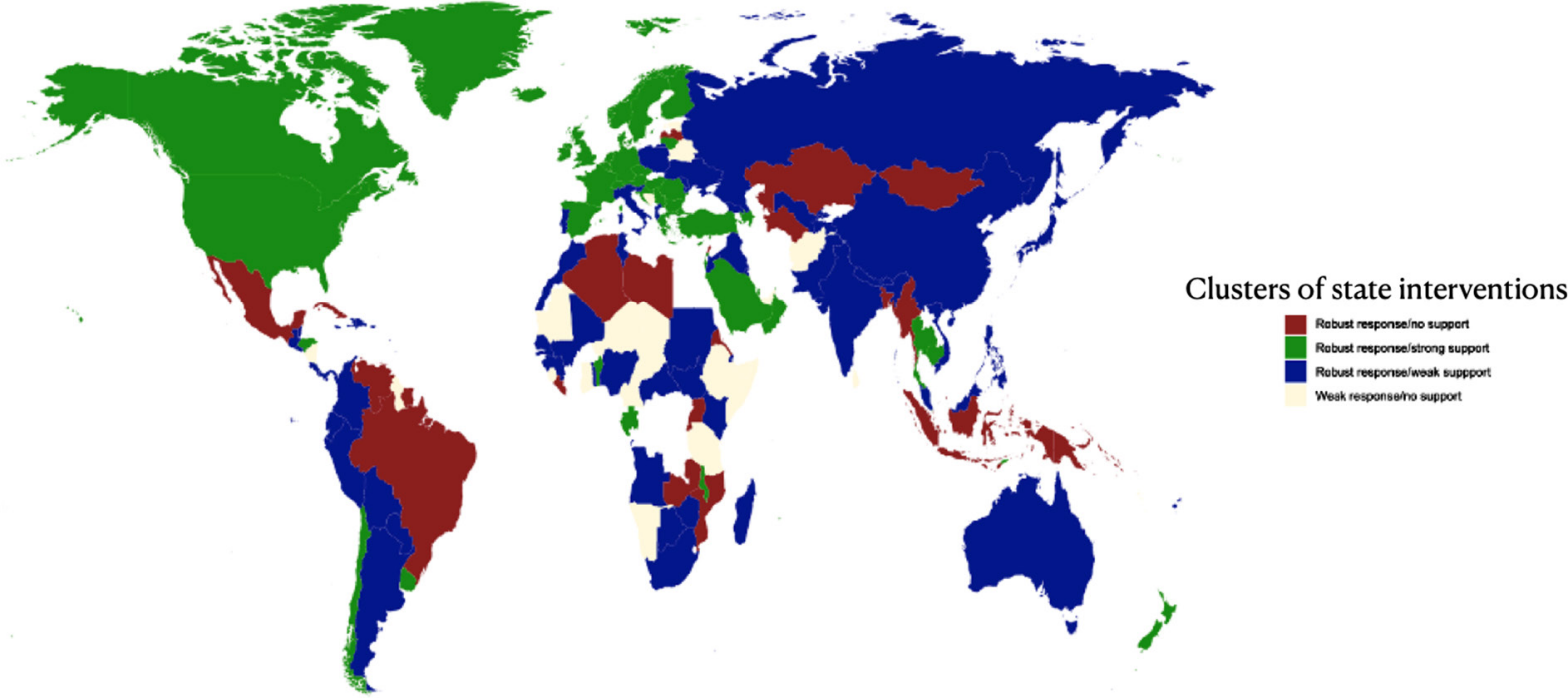
World heatmap of state interventions in the labor market (complete sample)

To illustrate the association between the clusters of state intervention and world systems, we visualized the clusters on a world heatmap in Figure 2 showing the demarcation between core, periphery, and semi‐periphery systems. Most of the core states combined generous income support measures alongside strict public health interventions, while the response in semi‐periphery state appears to be patchy, with lower levels of worker support. Periphery states afforded the lowest level of support with limited public health interventions.

**FIGURE 3:**
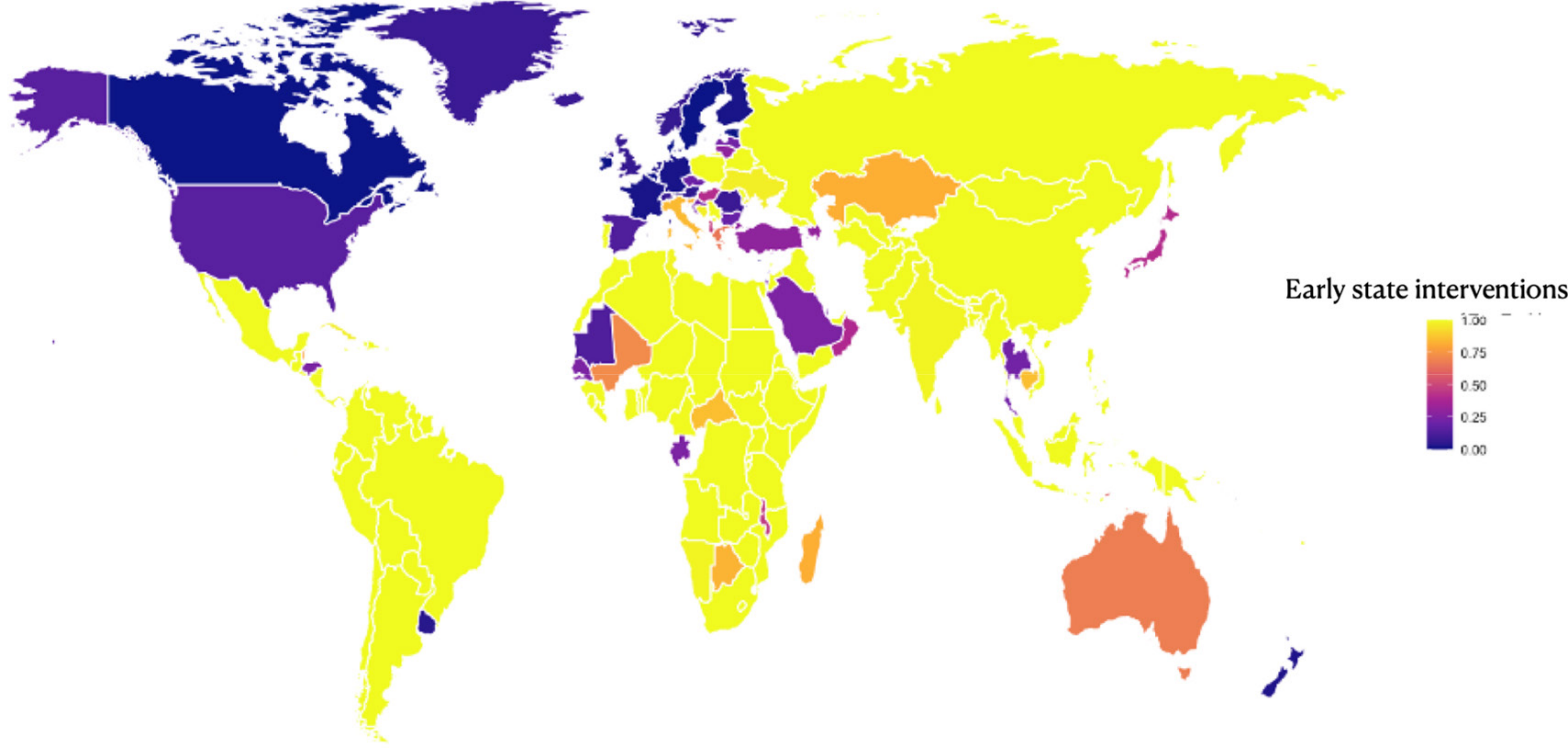
World heatmap of state interventions in the labor market (early interventions)

Next, we zoomed in on the early state interventions between March and June 2020, from the point in time when the World Health Organization declared a global pandemic through to the peak of the first wave. We estimated the combined share of states where income support was either non‐existent or covered less than 50 percent of the lost income. Figure 3 illustrates the distribution of this variable across world systems, again presented as a world heatmap (note, on this graph brighter colors correspond to delayed, less stringent interventions in the first months of the pandemic). Apart from demonstrating the gap between core, periphery, and semi‐periphery states, the graph reveals variation within world systems. For example, among core countries the United States, the United Kingdom, and Italy had less stringent interventions in the labor market at the onset of the pandemic. The same holds for Brazil relative to South Africa and India.

The established clusters and metrics signifying different trajectories of nation‐states’ interventions in the labor market were central to our econometric approach. We used interrupted time series (ITS) regression, a quasi‐experimental technique widely used to estimate the effects of population‐level policies or economic shocks. The COVID‐19 pandemic and subsequent state interventions are exogenous shocks that cause a sharp discontinuity in labor market outcomes that are unlikely to be explained by factors other than state interventions. The pre‐COVID period is easily identifiable and the use of national household panel surveys with waves before the pandemic enabled us to test for autocorrelation, seasonality, and white noise that can distort causal effects derived by ITS. To estimate comparative impacts of the pandemic across world systems, we employed a multi‐group interrupted time series design in line with Equation (1). Equation (1) is a linear probability model with individual fixed effects and standard errors clustered at country level.

(1) $$\begin{array}{c} Y_{\mathrm{icy}}=\beta_{0}+\beta_{1}Year+\beta_{2}Covid_{y}+\beta_{3}Year\cdot Covid_{y}+\beta_{4}WorldSystems+\beta_{5}WorldSystems\cdot Year\\ \;+\beta_{6}WorldSystems\cdot Covid_{y}+\beta_{7}WorldSystems\cdot Year\cdot Covid_{y}+\beta_{8}^{\prime }\cdot X_{\mathrm{icy}}+\alpha_{i}+\in_{\mathrm{icy}} \end{array}$$

In Equation (1) *i*, *c*, and *y* indexes individual, country, and year, respectively, where *i* = 1, 2, …, N and *c* = 1, 2, …, 17. *Year* captures yearly trends, and *Covid* is a dummy variable that captures the exogenous pandemic shock and is, therefore, equal to 1 (post‐intervention) or 0 otherwise (pre‐intervention). *Year Covid_y_* is an interaction term and indicates the slope change immediately after the pandemic. The key variable of interest is *WorldSystems Covid_y_* where β_6_ measures the change in the impact of the pandemic between the different world systems. β_7_ estimates the three‐way interaction effect and tests whether the world systems differ in post‐Covid‐19 slope changes. *X_icy_* includes country‐level control variables. Finally, *α_i_*, represents unobserved individual heterogeneity and ϵ_icy_ represents the idiosyncratic error term.

We estimated Equation (1) with the cluster of nation‐states derived by sequence analysis instead of world systems (see Equation (2)).

(2) $$\begin{array}{c} Y_{\mathrm{icy}}=\beta_{0}+\beta_{1}Year+\beta_{2}Covid_{y}+\beta_{3}Year\cdot Covid_{y}+\beta_{4}Sub‐groups+\beta_{5}Sub‐groups\cdot Year+\beta_{6}Sub‐groups\cdot Covid_{y}\\ \;+\beta_{7}Sub‐groups\cdot Year\cdot Covid_{y}+\beta_{8}^{\prime }\cdot X_{\mathrm{icy}}+\alpha_{i}+\in_{\mathrm{icy}} \end{array}$$

Lastly, we estimated the extent to which political populism, active labor market policies and collective bargaining affected early state interventions in the labor market by Equation (3) (where *Y_ct_* corresponds to the measurement of weak interventions at the onset of the pandemic shown in Figure 3).

(3) $$\begin{array}{c} Y_{\mathrm{ct}}=\beta_{0}+\beta_{1}\cdot Populism+\beta_{2}\cdot LaborPolicies+\beta_{3}\cdot CollectiveBargaining\\ \;+WorldSystemDummies+StatisticalControls+u_{\mathrm{ct}} \end{array}$$

In the next section, we report the outcomes of our regression analysis.

## FINDINGS

**FIGURE 4:**
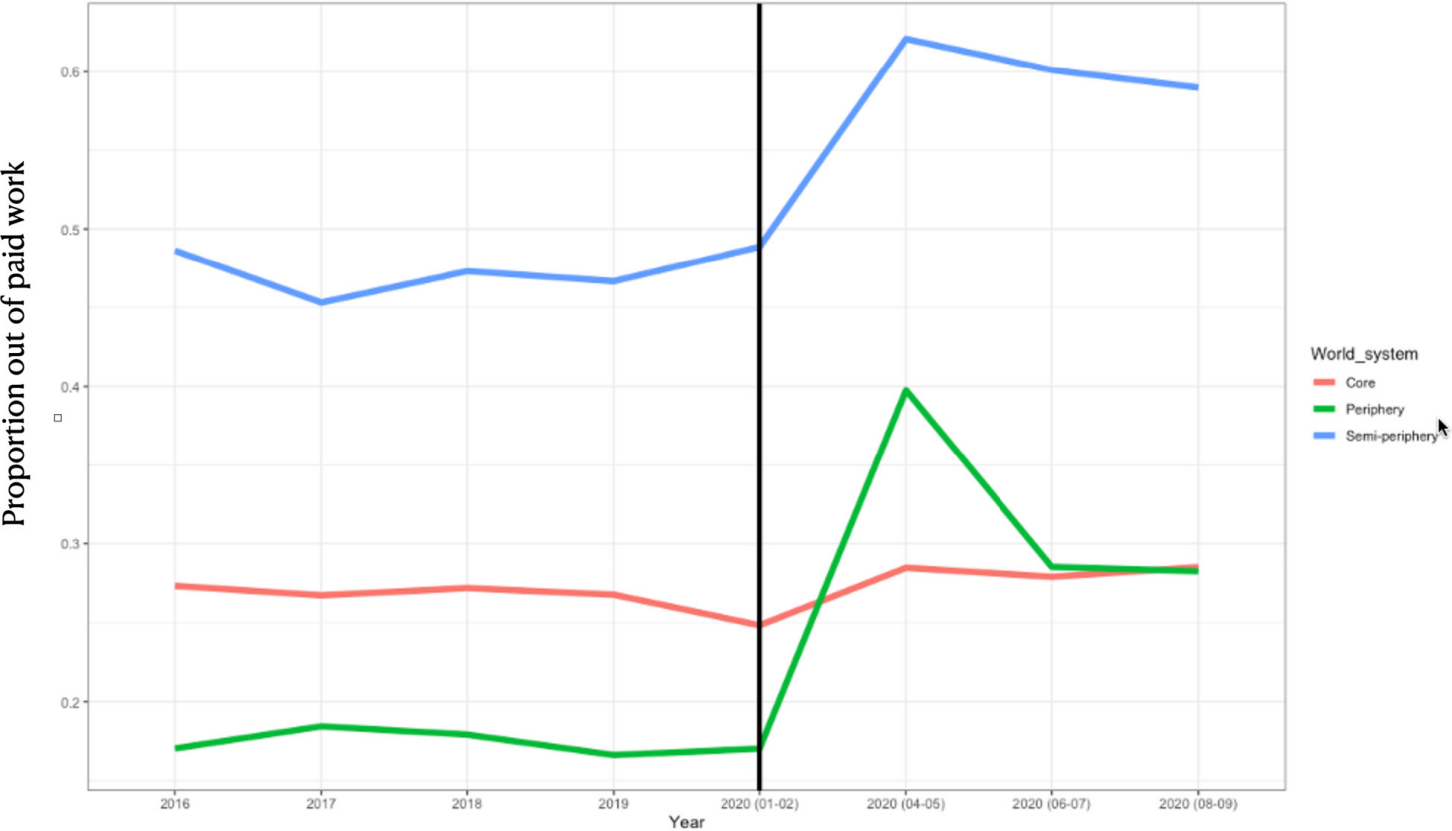
Discontinuity across world systems

The sharp discontinuity caused by the pandemic and its variation across world systems is depicted in Figure 4. Trends before the pandemic were similar between world systems and relatively smooth. The pandemic caused a sharp discontinuity increasing the likelihood of losing paid work. This is further supported by regression analysis reported in Table 2. We present models with different specifications including a pooled OLS regression in Model 1 and fixed effects with clustered standard errors in line with Equation (1) in Model 2. In both models, the causal effect is measured from the onset of the pandemic in February 2020 through to the peak of the first wave in June 2020 (narrowing this window to April–May 2020 had no material effect on regression estimates). The effects were sizable and statistically significant at less than 0.1 percent. In the robust fixed effects specification, the likelihood of dropping out of paid work at the beginning of the COVID‐19 pandemic was on average 19.2 percent higher in periphery states relative to core states and 6.1 percent higher among semi‐periphery states. Thus, the analysis so far corroborates Hypothesis 1.

**TABLE 2:** Regression coefficients for the causal effect of COVID‐19 crisis on the likelihood of losing paid work (core countries—omitted category)

	Estimates	Confidence intervals	*p*‐values	Estimates	Confidence intervals	*p*‐values
	(1)			(2)		
Covid‐19	0.006	0.003 to 0.009	<0.001	0.085	0.080 to 0.091	<0.001
World systems [Periphery]	−0.099	−0.108 to −0.090	<0.001	−3.806	−4.156 to −3.456	<0.001
World systems [Semi‐periphery]	0.203	0.201 to 0.205	<0.001	−1.234	−1.384 to −1.084	<0.001
Covid × World systems [Periphery]	0.252	0.239 to 0.266	<0.001	0.192	0.178 to 0.206	<0.001
Covid × World systems [Semi‐periphery]	0.133	0.129 to 0.137	<0.001	0.061	0.055 to 0.067	<0.001
Sample size	1,725,078					
R^2^ adjusted	0.052			0.064		
Individual controls	v			v		
Country controls	v			v		
Individual fixed effects				v		
Clustered standard errors				v		
Yearly trends	v			v		

**FIGURE 5:**
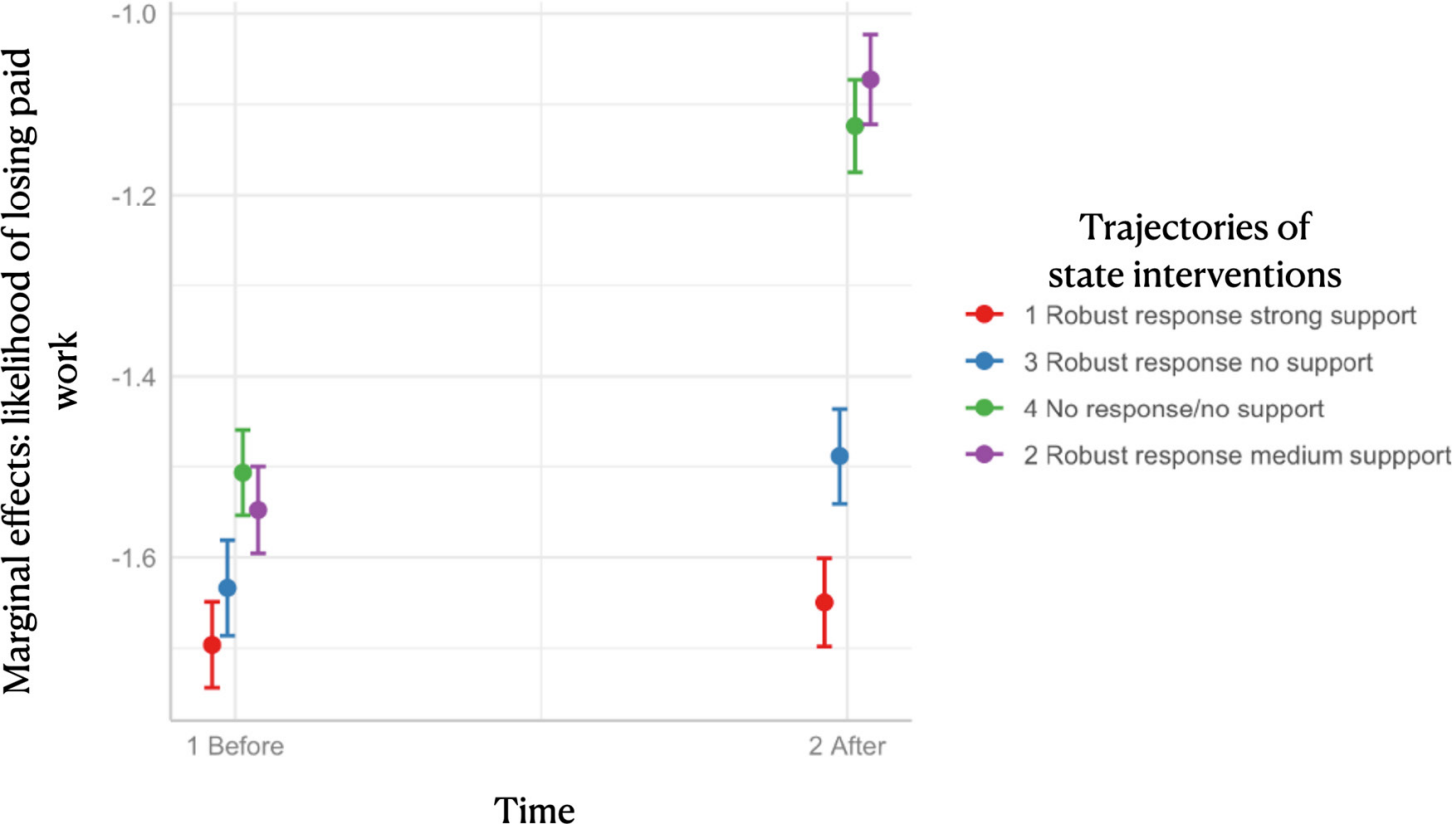
Causal effect of state interventions on the likelihood of dropping out of paid work across world systems

Table 3 reports regression outputs estimating the effect of state interventions in the labor market in line with Equation (2), while Figure 5 visualizes the respective causal effects with 95 percent confidence intervals. The table contains three models: Model 1 replaces world systems with the clusters of trajectories of state intervention in the labor market derived by sequence analysis estimating discontinuity at the cut‐off; Model 2 includes an extended post‐intervention period until Fall 2020; Model 3 uses the continuous measurements of the weakness of state intervention at the onset of the pandemic as a moderator to estimate the consequences of providing no or moderate‐income support in the first months of the pandemic in core states; and Model 4 provides the same specification as Model 3 but for periphery and semi‐periphery states only.

**TABLE 3:** Regression coefficients for the effects of trajectories of state interventions on likelihood of losing paid work (in models 1 and 2 stringent response and high‐income support—omitted category)

	Estimates	Confidence intervals	*p*‐values	Estimates	Confidence intervals	*p*‐values	Estimates	Confidence intervals	*p*‐values	Estimates	Confidence intervals	*p*‐values
	(1)			(2)			(3)			(4)		
Covid‐19	0.007	0.003 to 0.010	<0.001	0.086	0.081 – 0.091	<0.001	−0.163	−0.225 to −0.101	<0.001	−0.163	−0.225 to −0.101	<0.001
Weak response at the onset	—			—			0.836	0.623 to 1.049	<0.001	0.379	0.248 to 0.511	<0.001
Covid‐19 × Weak response at the onset							0.239	0.131 to 0.348	<0.001	0.720	0.621 to 0.819	<0.001
Trajectory state interventions [Stringent response‐moderate support]	0.305	0.301 to 0.309	<0.001	0.531	0.511 – 0.551	<0.001	—			—		
Trajectory state interventions [Stringent Response—no support]	0.193	0.191 to 0.195	<0.001	0.079	0.055 – 0.103	<0.001						
Trajectory state interventions [Weak Response—no support]	−0.028	−0.045 to −0.010	0.002	0.163	0.137 – 0.189	<0.001						
Covid‐19 × Trajectory [Stringent Response—moderate support]	0.085	0.075 to 0.095	<0.001	0.148	0.136 – 0.159	<0.001						
Covid‐19 × Trajectory [Stringent Response—no support]	0.137	0.133 to 0.141	<0.001	0.059	0.053 – 0.065	<0.001						
Covid‐19 × Trajectory [Weak Response—no support]	0.376	0.345 to 0.408	<0.001	0.297	0.266 – 0.328	<0.001						
Sample size	1,725,078						409,881			1,315,197		
R^2^ adjusted	0.055			0.064			0.042			0.109		
Individual controls	v											
Country controls	v											
Individual fixed effects	v											
Clustered standard errors	v											
Yearly trends	v											

These models provide a more nuanced account of the impact of state intervention on the likelihood of dropping out of paid work. Model 1 indicates a sharper discontinuity for nation‐states that deployed weak labor market interventions. Workers in nation‐states that had deployed less stringent public health interventions with almost no income support were 37.6 percent more likely to drop out of paid work at the onset of the pandemic relative to the states with strong income support policies, an effect that remained sizable in Fall 2020 as indicated by Model 2 (the effect size dropped to 29.7 percent). Nation‐states with robust response but different combinations of income support measures too suffered disproportionate effects. Stringent public health measures with no income support were associated with an increase in the likelihood of losing paid work by 13.7 percent relative to states with high‐income support. Moderate levels of income support reduced this gap to 8.5 percent in the first months of the COVID‐19 crisis, but, interestingly, exacerbated the impact on workers by elevating the likelihood of dropping out of paid work to 14.8 percent by Fall 2020.

One possible explanation for this effect is that state interventions in the labor market were particularly important at the onset of the pandemic while trajectories further down the line were affected by a combination of many other socioeconomic factors (for example, the longer‐term impact of the informal economy in periphery states). This is partially supported by the outcomes of Models 3 and 4 where weaker interventions in the labor market in the first months of the pandemic had a significant, sizable effect on the probability of losing paid work. Having no intervention at all between March and June 2020 would have increased the likelihood of losing paid work by 72 percent in semi‐periphery and periphery states compared to 23.9 percent in core states. Ultimately, a somewhat perplexing finding revealed in our analysis of state interventions requires further analysis in future research. Though overall it is clear that weaker labor market interventions had more negative effects compared with the cases where interventions were strongest.

The final set of regression estimates concern the determinants of the trajectories of nation‐states’ responses to the pandemic: political populism, active labor market policies, and collective bargaining coverage. Table 4 reports regression estimates in accordance with Equation (3) for two models: Model 1 is based on the OxCGRT aggregated database of 185 world countries; Model 2 shows the results when the measurements of populism, active labor market policies, and collective bargaining are merged with our 20‐country microdata. In both models, regression coefficients were sizable and statistically significant. We interpret the regression results by focusing on model one that covers a much wider range of countries than our household panel microdata and provide marginal effects for each hypothesized independent variable in Table 5. Table 5 contains predictions (i.e., predicted amount of time captured by the ratio scale from 0 to 1 when state interventions in the labor market were weak or non‐existent at the onset of the pandemic) for different values of independent variables—political populism, collective bargaining coverage and active labor market policies—alongside the corresponding 95 percent confidence intervals.

**TABLE 4:** Regression estimates for the determinants of weak state interventions at the onset of COVID‐19

Predictors	Estimates	Confidence intervals	*p*‐values	Estimates	Confidence intervals	*p*‐values
	Model (1)			Model (2)		
log (ALMPs)	−0.506	−0.707 to −0.305	<0.001	−0.616	−0.616 to −0.615	<0.001
log (Collective bargaining)	−0.075	−0.145 to −0.005	0.036	−0.100	−0.101 to −0.100	<0.001
log (Populism)	0.108	0.014 to 0.203	0.025	0.037	0.037 to 0.037	<0.001
Observations	215			1,725,078		
R^2^ adjusted	0.430			0.963		
Country‐level controls	v			v		
World systems dummies	v			v		

**TABLE 5:** Marginal effects for predictors of state interventions at the onset of the pandemic

Populism index	Political populism			Collective bargaining				Active labor market policies			
	Predicted	95% CI (LL)	95% CI (UL)	Collective bargaining coverage	Predicted	95% CI (LL)	95% CI (UL)	ALMPs index	Predicted	95% CI (LL)	95% CI (UL)
0.10	0.28	0.17	0.40	10%	0.52	0.42	0.63	20	0.92	0.69	1.19
0.30	0.38	0.31	0.46	30%	0.44	0.36	0.52	30	0.67	0.53	0.82
0.40	0.41	0.33	0.50	40%	0.42	0.33	0.51	40	0.51	0.42	0.61
0.50	0.43	0.34	0.53	50%	0.40	0.31	0.50	50	0.40	0.31	0.49
0.60	0.45	0.35	0.56	60%	0.39	0.29	0.49	60	0.31	0.22	0.41
0.70	0.47	0.36	0.59	70%	0.38	0.28	0.48	70	0.24	0.15	0.35
1.00	0.51	0.37	0.66	100%	0.35	0.24	0.47	80	0.19	0.08	0.30

According to Table 5, nation‐states with a higher level of populism (the populism index around 0.7 or higher) were likely to have limited or no worker support measures half of the time (on average) in the early months of the pandemic. This compares to less than 38 percent where the political discourse is not dominated by the right‐wing populists, a figure that according to our estimates can go as low as 17 percent (see the lower bound of 95% CI corresponding to the populism index = 0.1). To put these findings in a global context, the United States, Hungary, Philippines, and Brazil had some of the highest populist scores in the complete country database, between 0.8 and 0.95, in contrast to moderate (e.g., 0.3 in Portugal and Uruguay and 0.5 in India or South Africa) and low levels of populism (e.g., less than 0.1 in Germany, Denmark, New Zealand; slightly above 0.1 in Chile). Thus, controlling for economic and technological development, infection rates, and proximity to election, right‐wing populism could have led to a twofold increase in the likelihood of workers being unprotected at the beginning of the pandemic. This lends strong support to Hypothesis 3.

The effect of collective bargaining was less pronounced, albeit statistically significant and sizable. Table 5 reveals that the amount of time workers were poorly protected at the start of the pandemic could have been halved with an increase in collective bargaining coverage from 10 percent (e.g., the United States, Bulgaria, and Ethiopia) to 60 percent (e.g., Germany). The effect of ALMPs was also significant. Marginal effects reported in Table 5 suggest that where pre‐pandemic activation programs had been particularly strong (ALMP index ~0.8; e.g., Norway, Denmark, Singapore, Switzerland, and Austria) the amount of time when workers were poorly protected was reduced to a minimum. Where ALMPs were virtually non‐existent (ALMP index = 0.2 in Table 5; e.g., Venezuela, Nigeria, and Zimbabwe), workers were likely to remain poorly protected throughout the first months of the pandemic. This final set of regression estimates corroborate Hypotheses 4 and 5.

### Sensitivity analysis

Owing to the complex methodological design, we performed several sensitivity checks to ensure the robustness of our regression estimates. The fundamental assumption behind ITS is that the estimated causal effect unpicks a genuine discontinuity at the cut‐off. This depends on the characteristics of the time series in question: longitudinal data with seasonal trends and autocorrelated time series will produce misleading estimates because the counterfactual scenario (what would have happened in the absence of the COVID‐19 shock) will estimate the noise from a non‐stationary time series. To take these possibilities into account, we built an Auto‐Regressive Integrated Moving Average (ARIMA) model using time periods before the pandemic to train the model and produce predictions for the wave immediately following the pandemic. We used it as a counterfactual and re‐estimated our models with results conformable to those reported in the present study. Furthermore, in a multiple‐group ITS the assumption of parallel trends between the groups becomes critical. We estimated placebo interventions by arbitrarily choosing a wave before the pandemic and simulated an intervention. No such artificial interventions resulted in meaningful differences, whether we take world systems as a unit of analysis or individual countries. Lastly, the stable treatment value assumption could have been violated in the first months since the pandemic. This could have happened if workers protected by job retention schemes classified themselves as out of work. Although in such circumstances national surveys instructed respondents to classify themselves as in work, the possibility of a measurement error in the first post‐pandemic waves cannot be ruled out. In countries where additional waves were available until the beginning of 2021, we have rerun the models with longer post‐intervention periods. This did not affect the direction of causality and produced results similar to the main regression models.

## DISCUSSION AND CONCLUSIONS

We analyzed international inequalities in the impact of the COVID‐19 pandemic on participation in paid work across 20 countries representing three world systems: core, periphery, and semi‐periphery. The employment relations literature on the impact of Covid‐19 to date has primarily looked at OECD economies or provided robust econometric evidence for a single country (e.g., Adams‐Prassl et al., 2020; Galasso & Foucault, 2020; Kim et al., 2020; Koebel & Pohler, 2020). The present study is the first to offer an employment relations perspective on the emerging consequences of the pandemic on a global scale. Drawing on World Systems Theory, we found evidence that periphery and semi‐periphery states suffered a much sharper discontinuity in labor market participation at the onset of the pandemic, even though many periphery states did not experience an exponential growth of infections and deaths similar to core and semi‐periphery states. We causally linked this disparity to trajectories of state intervention in the labor market, demonstrating that they were influenced by right‐wing populism and the institutional foundations of labor markets proxied by collective bargaining and ALMPs.

Theoretically, this paper supports the utility of world systems theory as a global comparative framework. This is a novel contribution because to date the comparative employment relations literature has tended to frame analysis within the logic of Varieties of Capitalism or other institutional theories of advanced capitalist economies (Frege & Kelly, 2013; Grimshaw & Hayter, 2020; Streeck, 2016). We have highlighted the limitations of these theories for global analysis and demonstrated the value of WST in explaining the causal mechanisms underpinning the unequal effects of the pandemic. WST has been instrumental in hypothesizing the effects of political (right‐wing) populism and labor market institutions while also explaining heterogeneity within world systems. Right‐wing populism can take different forms in core and semi‐periphery (periphery) states, with a more pronounced cultural element in relation to the latter (Eichengreen, 2018). Yet, our study demonstrates a clear association between right‐wing populism and weaker state interventions. Fiscal constraints in periphery and semi‐periphery states could have played a part, amplifying the populist rhetoric at the onset and throughout the pandemic and delaying government interventions (Ghosh, 2020b). In core states, other factors including partisanship could have interacted with right‐wing populism to affect the early state response to the pandemic (Gitmez et al., 2020). Given the limitations of the pooled data used to analyze the effect of populism and the nature of the respective measurement, we were unable to unpick other political factors often associated with populist ideology including partisanship, extremism, and so on. The interplay between these factors is for future research.

Applying world systems theory enabled us to extend the effect of collective bargaining from core states, where it is highly expected (Adams‐Prassl et al., 2020; Johnstone et al., 2019), to semi‐periphery and periphery states where the role of formal voice channels and collective bargaining is deemed limited due to the prevalence of informal working arrangements (Grimshaw & Hayter, 2020). It is possible then that collective bargaining in the formal economy can have a spillover effect and increase the likelihood of earlier, more stringent state interventions in periphery and semi‐periphery states (Freeman, 2010; Hayter & Pons‐Vignon, 2018). Likewise, having ALMPs in place before the pandemic enabled nation‐states to act more swiftly to protect workers at the start of the COVID‐19 crisis. In semi‐periphery and periphery states, ALMPs could have provided some degree of protection to informal workers by shunting some of them (even if temporarily) into the formal sector (Dhingra & Kondirolli, 2021).

Looking forward, the comparative employment relations literature may utilize further the tenets of WST to account for a wider range of systemic inequalities between countries that are not native to the VoC framework. There are important lessons to be learned from governments’ handling of the new coronavirus pandemic (Dobbins, 2020). The consequences of COVID‐19 for semi‐periphery and periphery countries are often overlooked in comparative employment relations research. Our study serves as a reminder that COVID‐19 is a global pandemic where significant disparities occur not only within developed economies but between world systems too. The pandemic has revealed the willingness of core states to protect public health at the expense of an economic shock. Yet, not enough consideration appears to be given to the fact that it reverberates through the world system causing a devastating effect on workers in semi‐periphery and periphery states. Growing inequalities in public health outcomes prompted scientists to call for international cooperation to combat the immediate and long‐term ramifications of the current and future pandemics (Brown & Susskind, 2020; Momtazmanesh et al., 2020). Our study supports the need for a similar approach to protect workers vulnerable to such global shocks.

Our study employed a robust quasi‐experimental analysis of household panel microdata. However, it has not escaped limitations. Many country samples were derived via online questionnaires distributed to smaller subsets of original panels. The longer‐term effects of COVID‐19 and state interventions in the labor market will become more transparent when full editions of national household panel surveys are commissioned and made available for academic research. Future studies may also consider expanding the geographical scope of core, periphery, and semi‐periphery states to include Eastern Europe, Russia, and Southeast Asia. This will enable a more critical engagement with world systems theory.

Lastly, while our study focused on participation in paid work, future research may consider additional outcomes, for example, the trade‐offs between employment and working hours that were significant in core countries. Given the number of countries included in our study, we were unable to provide fine‐grained estimates separately for employed, self‐employed, and informal workers. While our econometric specification is robust to such disparities between world systems, future studies may use a smaller subset of countries to investigate the extent to which trajectories of state intervention disproportionately affected such groups of workers.
